# Draft genome sequence of two “*Candidatus* Intestinicoccus colisanans” strains isolated from faeces of healthy humans

**DOI:** 10.1186/s13104-023-06447-3

**Published:** 2023-08-17

**Authors:** Joyce Zhou, Joel A Boyd, Bozica Nyeverecz, Charlotte Vivian, Nicola Angel, David L A Wood, Philip Hugenholtz, Gene W Tyson, Lutz Krause, Páraic Ó Cuív

**Affiliations:** 1 Microba Life Sciences, Queensland, Brisbane, Australia; 2 Australian Centre for Ecogenomics, School of Chemistry and Molecular Biosciences, The University of Queensland, St Lucia, QLD Australia

**Keywords:** Human, Gut, Health, Microbiome, Uncultured, Intestinicoccus colisanans

## Abstract

**Objectives:**

In order to provide a better insight into the functional capacity of the human gut microbiome, we isolated a novel bacterium, “*Candidatus* Intestinicoccus colisanans” gen. nov. sp. nov., and performed whole genome sequencing. This study will provide new insights into the functional potential of this bacterium and its role in modulating host health and well-being. We expect that this data resource will be useful in providing additional insight into the diversity and functional potential of the human microbiome.

**Data description:**

Here, we report the first draft genome sequences of “*Candidatus* Intestinicoccus colisanans” strains MH27-1 and MH27-2, recovered from faeces collected from healthy human donors. The genomes were sequenced using short-read Illumina technology and whole-genome-based comparisons and phylogenomics reconstruction indicate that “*Candidatus* Intestinicoccus colisanans” represents a novel genus and species within the family Acutalibacteraceae. Both genomes were estimated to be > 98% completed and to range in size from 2.9 to 3.3 Mb with a G + C content of approximately 51%. The gene repertoire of “*Candidatus* Intestinicoccus colisanans” indicate it is likely a saccharolytic gut bacterium.

**Supplementary Information:**

The online version contains supplementary material available at 10.1186/s13104-023-06447-3.

## Objective

The healthy human gut is colonized by a diverse microbial community that provides a suite of functionalities relevant to host health. It is estimated that over 70% of the human microbiome remains uncultured and this remains a key challenge to better understanding the ecological and functional role of individual microbial species [1]. To better address this challenge, we applied a genome-directed isolation approach [2] to isolate a novel uncultured bacterium that is both numerically abundant and prevalent in the healthy human gut. To isolate “*Candidatus* Intestinicoccus colisanans” MH27-1, a dilution-to-extinction enrichment culture series was generated from a faecal sample collected from a healthy human donor and incubated at 37 °C. Following metagenomic sequencing, a low diversity enrichment culture dominated by “*Candidatus* Intestinicoccus colisanans” MH27-1 was identified. “*Candidatus* Intestinicoccus colisanans” MH27-1 was isolated on YCFA medium supplemented with 5% v/v of an aqueous faecal extract and 1.5% w/v agar following incubation at 37 °C. As “*Candidatus* Intestinicoccus colisanans” was uncultured, we hypothesized that the aqueous faecal extract was necessary for growth. However, “*Candidatus* Intestinicoccus colisanans” MH27-1 grew after 72 h of culture in PYG broth medium at 37 °C thereby revealing the aqueous faecal extract was dispensable for growth. To isolate “*Candidatus* Intestinicoccus colisanans” MH27-2, a dilution-to-extinction enrichment culture series was generated from a faecal sample collected from an independent healthy human donor incubated at 37 °C. A low diversity enrichment culture dominated by “*Candidatus* Intestinicoccus colisanans” MH27-2 was identified following metagenomic sequencing. Following purification on PYG medium supplemented with 1.5% w/v agar at 37 °C, an axenic isolate was produced that grew after 72 h of culture in PYG broth medium at 37 °C. Both isolates formed raised creamy white/milky colonies with an entire margin on agar and were typically observed as Gram-variable coccoid/ovoid cells that were often present as pairs or short chains (see Supplementary Information Figures S1 and S2).

## Data description

We performed whole-genome sequencing to assess the functional potential of “*Candidatus* Intestinicoccus colisanans” and better understand its interactions with the host. Both strains were grown in PYG based medium and DNA was extracted using the Nextera DNA Flex Microbial Extraction protocol [3]. DNA libraries were prepared using the Illumina DNA Prep Library Preparation Kit as per the manufacturer’s instructions, with unique dual indexes (IDT for Illumina DNA/RNA UD Index set A-D 20027213-6) and PhiX spike in at 2%, and sequenced on the NovaSeq6000 in 2 × 150 bp format. The libraries produced 5,719,975 and 3,565,993 150 bp paired-end reads for “*Candidatus* Intestinicoccus colisanans” MH27-1 and MH27-2, respectively. For QC, assembly and functional annotation, default parameters were used for software except where otherwise noted. Illumina reads were trimmed and quality controlled using Trimmomatic v0.36 (ILLUMINACLIP:adapters_NexteraPE-PE_TruSeq3-PE.fa:2:30:10, LEADING:3, TRAILING:3, SLIDINGWINDOW:4:15, CROP:150 HEADCROP:0, MINLEN:100) [4], and PhiX reads were removed using bbduk from bbmap v38.68 [5]. Reads were merged using using bbmerge from bbmap assembled using Spades v3.13.0 [6]. Quality controlled reads were merged assembled producing 31 contigs for “*Candidatus* Intestinicoccus colisanans” MH27-1 (N50 = 308,050) and 25 contigs for MH27-2 (N50 = 454,909).

**Table 1:** Overview of data files/data sets

Label	Name of data file/data set	File types (file extension)	Data repository and identifier (DOI or accession number)
*Data file 1*	*MH27-1 and MH27-2 NCBI BioProject*	No file format	NCBI BioProject Database https://identifiers.org/ncbi/bioproject:PRJNA7795 [13]
*Data file 2*	*MH27-1 Illumina raw sequences*	*fastq*	NCBI Sequence Read Archive https://identifiers.org/ncbi/insdc.sra:SRX13161736 [14]
*Data file 3*	*MH27-1 whole genome assembly sequence*	*fasta*	NCBI Assembly Database https://identifiers.org/ncbi/assembly:GCA_021029585.1 [15]
*Data file 4*	*MH27-2 Illumina raw sequences*	*fastq*	NCBI Sequence Read Archive https://identifiers.org/ncbi/insdc.sra:SRX13161735 [16]
*Data file 5*	*MH27-2 whole genome assembly sequence*	*fasta*	NCBI Assembly Database https://identifiers.org/ncbi/assembly:GCA_021029595.1 [17]

The genome size of “*Candidatus* Intestinicoccus colisanans” MH27-1 was 3,304,406 bp (GC = 51.2%) and candidatus “*Candidatus* Intestinicoccus colisanans” MH27-2 was 2,969,717 bp (GC = 50.9%). Both genomes were estimated to be 98.66% complete and 0% contaminated by CheckM v1.0.18 [7]. NCBI designated the new isolates as Oscillispiraceae sp. however standardised genome-based taxonomy using the Genome Taxonomy Database r89 [8] assigned both isolates to the uncultured bacterial species *UBA1417 sp003531055* (GTDB taxonomy: d__Bacteria; p__Bacillota_A; c__Clostridia; o__Oscillospirales; f__Acutalibacteraceae; g__UBA1417; s__UBA1417 sp003531055). Analysis with Prokka v1.14.6 [9] revealed “*Candidatus* Intestinicoccus colisanans” MH27-1 and MH27-2 encoded 3151 and 2789 protein coding genes, respectively. plasmidSpades (v3.15.3) analyses, followed by manual curation, revealed a small plasmid in both MH27-1 (5.3 kb; 56.8% GC; 6 proteins) and MH27-2 (6.1 kb, 50.1% GC; 8 proteins). Analysis with Phaster [10] identified a 31Kb putative prophage (GC 52.2%) containing 44 proteins in “*Candidatus* Intestinicoccus colisanans” MH27-1.

Gapmind [11] analysis revealed both strains encode complete pathways for the biosynthesis of 7 amino acids (arg, asp, cys, gly, glu, lys and val). Analysis with dbCAN2 revealed MH27-1 and MH27-2 encode 44 and 45 carbohydrate active enzymes respectively, including four copies of GH5 (cellulase) and GH29 (fucosidase) enzymes each. Analysis with AntiSmash v5.1.2 [12] revealed “*Candidatus* Intestinicoccus colisanans” MH27-1 and MH27-2 encode one and two cryptic RiPP biosynthetic gene clusters, respectively.

In summary, there is a renewed interest in applying improved culture-based approaches to isolate novel gut microbes (reviewed by [18]). The isolation of “*Candidatus* Intestinicoccus colisanans” will enable a more thorough evaluation of its role in health and disease, and a mechanistic dissection of its functional capacities.

## Limitations

The genome sequences of “*Candidatus* Intestinicoccus colisanans” MH27-1 and MH27-2 were produced from short read data and remain incomplete. The closure of these genomes coupled with the isolation and sequencing of additional strains will provide a greater insight into the gene repertoire and functional capacity of this taxon.

### Electronic supplementary material

Below is the link to the electronic supplementary material.

Supplementary Material 1

## Data Availability

The data described in this Data note can be freely and openly accessed on Genbank under the accession numbers GCA_021029585.1 and GCA_021029595.1 (biosample numbers SAMN23040991 and SAMN23040992). Please see Table 1 for details and links to the data. The isolates were deposited as *Intestinicoccus colisanans* MH27-1 and MH27-2 with the National Measurements Institute (Australia) culture collection under accession numbers V21/015887 and V21/015888, respectively.

## Declarations

### Experimental methods

All experiments were performed in accordance with relevant Queensland and Australian governmental guidelines and regulations.

## Abbreviations

GC: Guanine–Cytosine content
GH: Glycoside hydrolase
RiPP: Ribosomally synthesized and post-translationally modified peptide
