# Supplementary material for: Draft genome sequence of two “Candidatus Intestinicoccus colisanans” strains isolated from faeces of healthy humans
Source: BMC Res Notes. 2023 Aug 17;16:174. doi: 10.1186/s13104-023-06447-3 (PMC10433555; doi:10.1186/s13104-023-06447-3)
Supplement: Supplementary file 1 — Supplementary Material 1 [file 13104_2023_6447_MOESM1_ESM.docx]

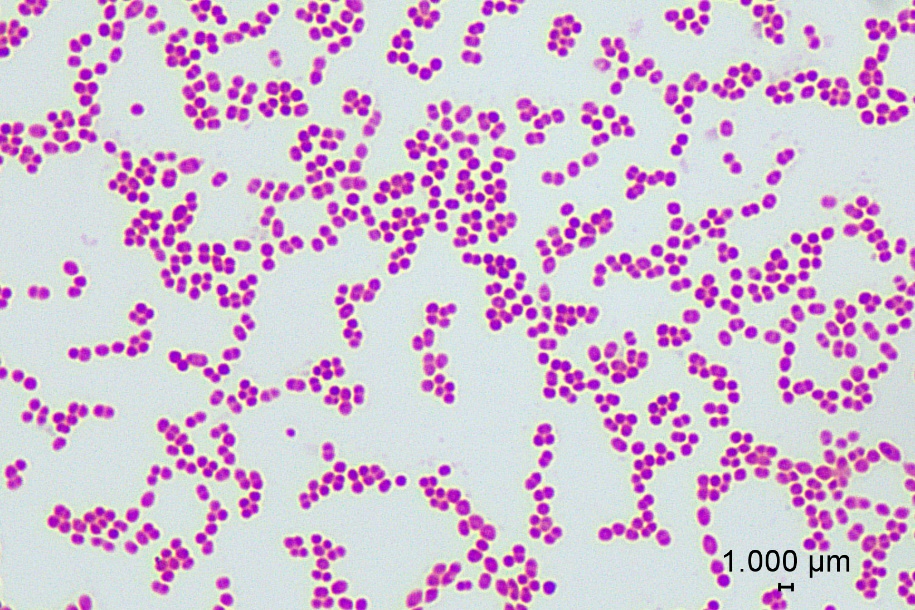


Figure S1. “*Candidatus* Intestinicoccus colisanans” MH27-1 predominantly stains Gram negative and is typically observed in pairs or short chains when grown in broth.


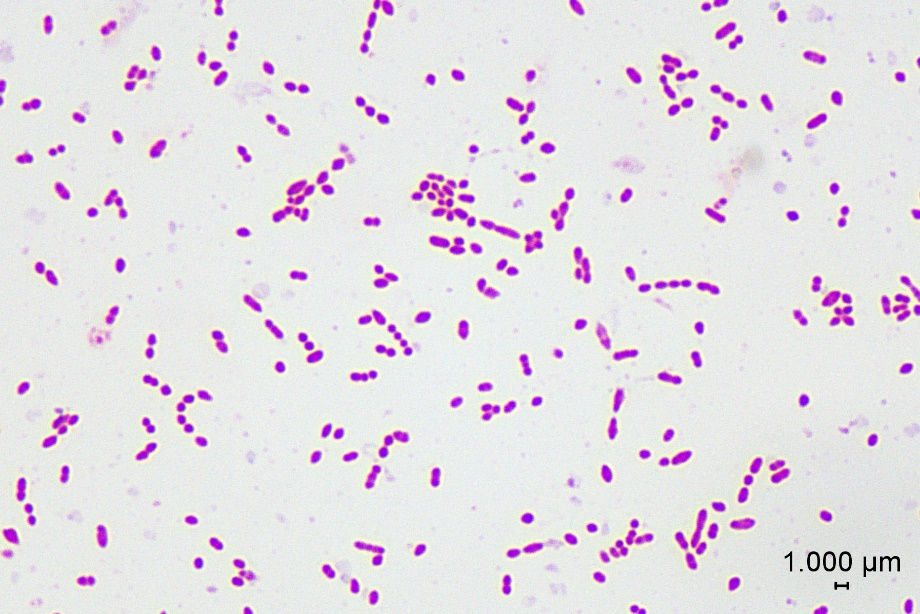


Figure S2. “*Candidatus* Intestinicoccus colisanans” MH27-2 predominantly stains Gram negative and is typically observed in pairs or short chains when grown in broth.
